# A critical appraisal of MAO-B inhibitors in the treatment of Parkinson’s disease

**DOI:** 10.1007/s00702-022-02465-w

**Published:** 2022-02-02

**Authors:** Wolfgang H. Jost

**Affiliations:** Parkinson-Klinik Ortenau, Kreuzbergstr. 12-16, 77709 Wolfach, Germany

**Keywords:** MAO-B inhibitors, Selegiline, Rasagiline, Safinamide, Parkinson’s disease

## Abstract

Since the 1980s, the MAO-B inhibitors have gained considerable status in the therapy of the Parkinson’s disease. In addition to the symptomatic effect in mono- and combination therapies, a neuroprotective effect has repeatedly been a matter of some discussion, which has unfortunately led to a good many misunderstandings. Due to potential interactions, selegiline has declined in significance in the field. For the MAO-B inhibitor safinamide, recently introduced to the market, an additional inhibition of pathological release of glutamate has been postulated. At present, rasagiline and selegiline are being administered in early therapy as well as in combination with levodopa. Safinamide has been approved only for combination therapy with levodopa when motor fluctuations have occurred. MAO-B inhibitors are a significant therapeutic option for Parkinson’s disease, an option which is too often not appreciated properly.

## Introduction

MAO-A (*Monoamine oxidase inhibitor-B*) inhibitors are used in anti-depressive therapy and MAO-B inhibitors in Parkinson therapy. At present, the MAO-B inhibitors selegiline, rasagiline and safinamide have been approved for treatment of Parkinson’s disease. Clinical studies were initiated in the 1970s (Knoll 1978; Riederer et al. 1978), with the original idea of Riederer and Youdim (Tábi et al. 2020). Selegiline was then introduced in the 1980s and 20 years later rasagiline internationally. Finally, safinamide was approved in numerous countries and launched on the market as of 2015.

## Selegiline

The first description of a levodopa-reinforcing or levodopa-reducing effect appeared in the 1970s, the decade when the first approval for levodopa preparations was granted in combination with a decarboxylase inhibitor (Birkmayer et al. 1985; Knoll 1978). Selegiline, also known since its introduction as l-Deprenyl, has been approved for mono- and combination therapies.

Selegiline is a relatively selective, irreversible inhibitor for central MAO-B. The half-life period of the substance ranges around 40 h, and this short period distinguishes it clearly from the extremely long half-life of 40 days in the case of cerebral MAO-B. This longer half-life in turn results from a neosynthesis of the enzyme because selegiline is an example of a so-called “suicide” inhibitor (Fowler et al. 1987, 1994). And precisely, this long time of recovery should be taken into account when administering selegiline with other drugs in PD patients.

Selegiline is rapidly reabsorbed and demonstrates a strong plasma–protein binding. Due to a high degree of lipophilicity, the blood–brain barrier can easily be overcome. Studies have shown that after intravenous administration, a maximum concentration can already be measured in the striatum after only five minutes (Fowler et al. 1987]. A maximum in plasma concentration is reached after 30 to 120 min. The substance demonstrates a high first-pass effect and is mainly metabolized in the liver (probably the cytochrome-P450-system) and eliminated renally.

**Fig. 1:**
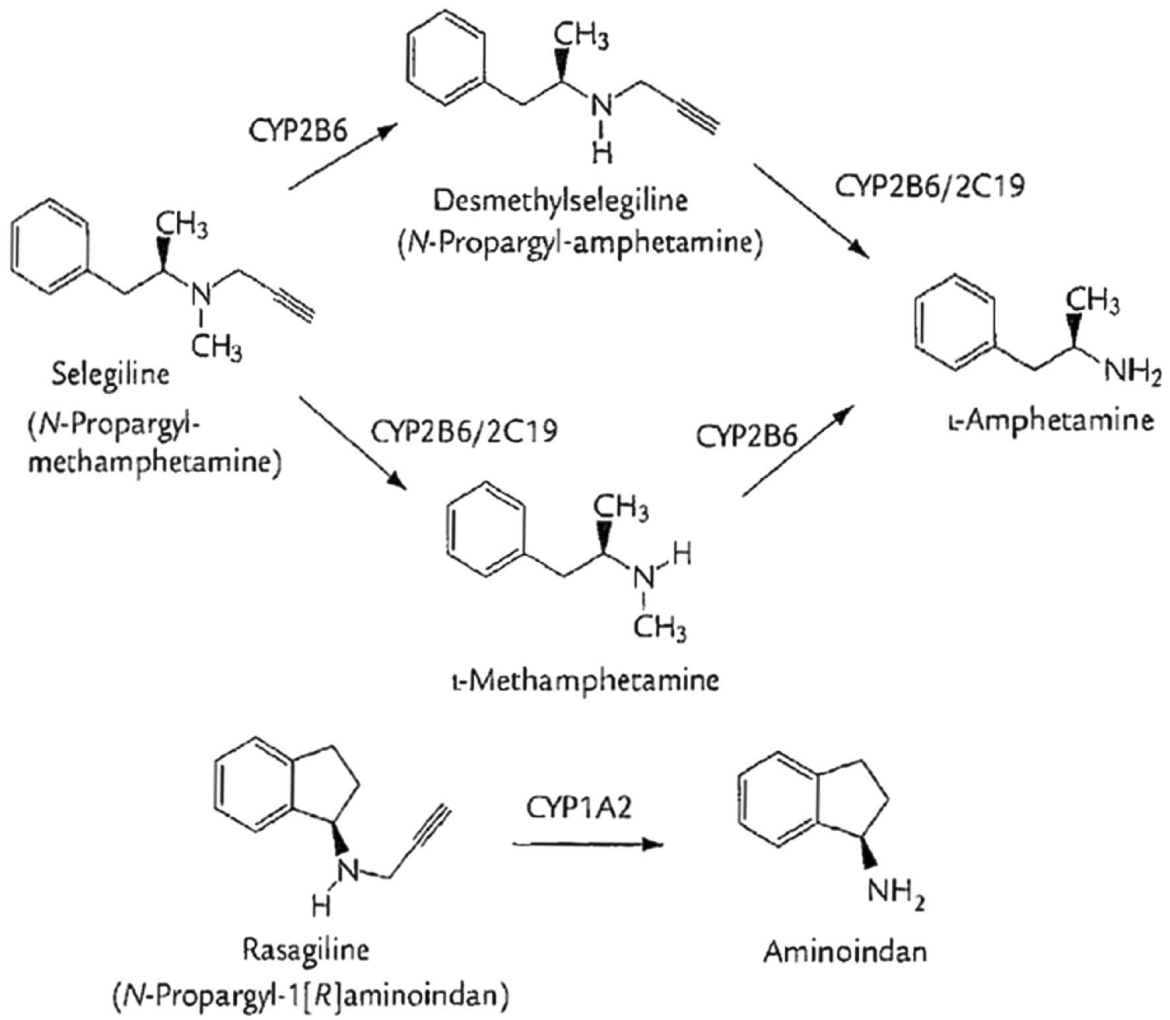
Metabolism of selegiline and rasagiline (after Chen et al. 2007)

Metabolisation proceeds over methamphetamine to amphetamine whereby under therapeutic doses no relevant amphetamine effect is to be expected (Fig. 1).

As a further preparation, a dissolving tablet was also available. This preparation was given at a lower dose, and the switch from traditional selegiline proved unproblematical (Ondo et al. 2011). A dissolving tablet (lyophilisate) contains 1.25 mg of selegiline HCL and corresponds to 10 mg in the usual selegiline tablet form (comparative plasma concentrations) (Clarke et al. 2003; Saeger 1998). The advantage of the sublingual application lies in by-passing first-pass metabolism and thus in avoiding amphetamine derivates. At present, the preparation is not available.

### Mode of action of selegiline

The first step in increasing cerebral levodopa concentrations was to inhibit decarboxylase. Thus, the attempt was made, in a second step, to inhibit the cerebral metabolism of levodopa by means of extra- and intraneuronal MAO. This idea was largely driven by Peter Riederer (Riederer et al. 1978). Clinical research succeeded with selegiline (deprenyl). Under the use of this selective MAO-B inhibitor, the dopamine concentrations were increased in the synaptic gap. In addition, a weak inhibition of dopamine uptake occurs.

To achieve the pharmacodynamic effect, the MAO-B has to be inhibited by at least 80% (Green et al. 1977). In therapeutic doses (10 mg), MAO-B is completely inhibited in thrombocytes. This then is the rationale for deciding on the routine dosage. The recommendation of using 1 mg of selegiline per 10 kg of body weight is relatively arbitrary and has not been scientifically corroborated. In principle, the decision for five or 10 mg is not based on scientific data (LeWitt 2009).

### Clinical effects of selegiline

Selegiline has been approved for both mono- and combination therapies. The symptomatic effect of monotherapy can be assessed as low to moderate, but the substance achieves a better rating in combination with levodopa. In that case, selegiline demonstrates, among others, a levodopa-smoothing effect and is beneficial for treating end-of-dose akinesia. The usual dose is 10 mg/die, given in either one or two portions. Higher doses are not recommended (see above).

In a recent study, the UPDRS score improved under selegiline monotherapy by 6.26 ± 7.86 (versus 3.14 ± 6.98 with a placebo) (Mizuno et al. 2017). In addition to the symptomatic effect, both a levodopa-saving effect and a positive effect on late motor complications have been described for selegiline (Dashtipour et al. 2015). There are also long-term studies which describe an increased rate of dyskinesia, which may best be explained by the dopaminergic effect (Shoulson et al. 2002). Freezing phenomena is said to occur less frequently (Shoulson et al. 2002; Zhang et al. 2016).

Data on the amount of levodopa saved differ strongly. Most studies claim an average savings effect of 20 und 30%. It is noteworthy that higher savings are seen especially in open studies. Here the overall dosage has to be taken into consideration. A saving of 20% under an overall dose of 300 mg of levodopa has to be seen as substantially different when compared to an overall dose of 1000 mg.

A double-blind study by Myllylä et al. (1997) of 5 years duration was able to demonstrate that without selegiline 725 mg of levodopa were necessary, while with selegiline only 405 mg of levodopa were required, that is, 45% less. In this study, the effect even increased over time (Myllylä et al. 1997).

Various publications from the DATATOP study (PSG 1989a, b, 1993, 1998) have frequently been cited. The most relevant finding was that with the administration of selegiline, giving levodopa can be delayed by several months (PSG 1989a, b, 1993, 1998, Tertrud and Langston 1989). But the conclusion that a neuroprotective effect is at work has to be viewed critically.

The effect of selegiline has been described as most noticeable for the first year (PSG 1989a) and as declining in long-term use (Rinne 1987; Yahr 1989). This has led to the frequent conclusion that selegiline should only be administered in the initial stage of the disease. But the results from Myllylä et al. (1997) as well as those from the SELEDO study (Przuntek et al. 1999) and one from Pålhagen et al. (2006) are in clear contradiction here. There is no decline over time. In addition, being convinced that the substance does have a neuroprotective effect automatically implies that its discontinuation in the course of treatment does not make sense. The long-term study by Mizuno et al. (2019) exhibited the best results after 20 weeks, whereby however the side effects were relatively strong (44.3%), and only 67.9% of the patients could be treated for more than 56 weeks (while in the control phase, tolerability was very good (Mizuno et al. 2017)).

In this context, a publication from Larsen et al. (1999) should be mentioned: In this double-blind study extending five years, the levodopa savings (of 424 vs. 506 mg/day) and in addition a milder course of the disease could be demonstrated. The mild course became even more apparent with time and persisted after discontinuation of selegiline. This effect cannot simply be explained by the slight symptomatic effect and may perhaps be an indication of a neuroprotective effect.

In therapeutic, but especially in over-therapeutic doses, an antidepressant effect has been observed, but is not covered by sufficient study data.

### Adverse effects of selegiline

The most frequent adverse effect is insomnia which particularly occurs when the substance is given in the late afternoon or the evening. For this reason, drug administration is recommended for the morning or at noon post-prandial. In combination with levodopa, confusional states and hallucinations can occur, and in addition, levodopa-induced hyper- or dyskinesias can be aggravated.

As opposed to the d-form, the therapeutically used l-form has no relevant amphetaminergic effect. Potential for addiction does not exist for selegiline (Yasar et al. 1996).

### Interactions of selegiline

One interaction holds for tyramine. The much-feared “cheese-effect” (which can, among others, induce hypertensive crises) is not to be expected under therapeutic doses. At most a minor amplification occurs in the sympathicomimetic effect of tyramine.

Unfortunately, selegiline exhibits further medicinal interactions (Csoti et al. 2012). The substance should not be given with sympathomimetic drugs, dampening neuropharmacological drugs and pethidin.

Caution is further recommended when jointly combining the substance with SSRI (serotonin reuptake inhibitors). The risk thereby is admittedly slight, but in combination with SSRI a reinforcement of the serotonergic effect can occur. Before giving selegiline, SSRI should be discontinued at the least 2 weeks beforehand, and in the case of fluoxetine by even as much as 5 weeks. In addition to SSRI, selegiline should not be combined with MAO-A inhibitors, triptans (in particular rizatriptan) and sibutramine.

In their material on technical information, the manufacturers make mention of numerous contraindications when combining selegiline with levodopa.

- hypertonia
- hyperthyreosis
- phaeochromocytoma
- narrow angle glaucoma
- prostatic hyperplasia with residual urine
- tachykardia
- cardiac arrhythmias
- angina pectoris
- psychosis
- dementia

### Contraindications for selegiline

Contraindications are listed for: severe hypertonia, cardiac arrhythmias and severe angina pectoris. Patients who have had an exogenic psychosis or who have extensive cerebral damage or advanced dementia should not be treated with selegiline. Further contraindications include patients with gastric or duodenal ulcers.

## Rasagiline

Rasagiline (*N*-Propargyl-1-[R]-aminoindan) is a reversible, selective MAO-B inhibitor (Finberg et al. 1996) and has five to ten times stronger MAO-B inhibition than selegiline (Finberg et al. 1996; Youdim et al. 2001). Its bioavailability reaches approximately 36% and is not compromised in any way by food ingestion. Metabolisation occurs mainly in the liver, and the metabolite is aminoindane, not amphetamine derivates (see Fig. 1). The precise significance of aminoindane has not yet been clarified (Müller and Reichmann 2012).

In the meantime, a number of studies have been published on rasagiline, some of which extended over several years (Hauser et al. 2016; Jankovic et al. 2014; Jiang et al. 2020; Lew et al. 2010; PSG 2002; Peretz et al. 2016; Rascol et al. 2005, 2016; Stern et al. 2004), notably: TEMPO, PRESTO, LARGO und ADAGIO:

The TEMPO study (Hauser et al. 2016; PSG 2002), which included 404 patients, aimed at identifying the effect and the safety of the medication in monotherapy. A distinct improvement in motor symptoms could be demonstrated for both the 1 mg and the 2 mg dose. The study extension proved interesting: Here all the patients, even the placebo group, received rasagiline (PSG 2004). After a year, the two groups differed profoundly, showing a decided advantage for the patients who had been given rasagiline from the very start. In the course of the trial period, just short of half, the patients received monotherapy after 2 years, 23% after four years and 13% after 6 years (Lew et al. 2010).

LARGO (Rascol et al. 2005) and PRESTO (PSG 2005) are studies which clearly demonstrated that rasagiline shows a good effect in combination therapy as well (for example with an increase in on-time). The LARGO study was able to show that the effects of rasagiline and entacapone are comparable (Rascol et al. 2005). According to these studies, the savings effect for levodopa in the case of rasagiline is higher than for selegiline.

The ADAGIO study (Hauser et al. 2016; Jankovic et al. 2014; Olanow et al. 2009; Rascol et al. 2011), a prospective study with delayed-start design, enrolled 1176 patients who had been diagnosed with Parkinson’s disease for an average of four-and-a-half months (SD ± 4.6) and had an UPDRS score of 20.4 (SD ± 8.5). The study extended over a period of 72 weeks and was able to demonstrate that with 1 mg of rasagiline the clinical course was significantly better, even after the placebo group had been given rasagiline after 36 weeks. The group with 2 mg failed to reach the study objective. There was considerable speculation on the reasons for this effect, and finally the so-called “floor effect” was favored, indicating that the diagnostic sensitivity of the UPDRS scale was insufficient in the lower range of the scale. 683 patients were kept under further observation. Unfortunately, there was no significant difference between the two groups (Rascol et al. 2016).

Rasagiline has a symptomatic mode of treatment in the early as well in the later stages (McCormack 2014). Clinical experience reveals that a major percentage of patients profits from administering rasagiline in vigilance, mood and quality of life. A rather large open study has substantiated these observations (Jost et al. 2008). In another study (Barone et al. 2015), the effect of rasagiline on depression in PD patients is less clear: At week 4, there was a significant difference in favor of rasagiline, while at week 12, rasagiline did not differ from placebo in improving depressive symptoms, as assessed by the Beck Depression inventory scale. These studies demonstrated improvements not only in motor behavior and non-motor disturbances, but also importantly in cognition and other non-motor aspects (Hanagasi et al. 2011; Olanow et al. 2009). One study that targeted changes in cognitive functioning under rasagiline however could not demonstrate any improvement (Weintraub et al. 2016). Rasagiline has been administered successfully in older patients (Tolosa and Stern 2012). In a study on preladenant, rasagiline was selected as the active comparison, and interestingly neither of the two substances, not even rasagiline, was superior to the placebo (Stocchi et al. 2017).

Some patients may experience a delay in the onset of action which means that a final evaluation of the therapy can only be done after up to four weeks of the intervention.

The tolerability of rasagiline is categorized as good (Jiang et al. 2020), and the adverse reactions correspond to those of selegiline (both substances are irreversible MAO-B inhibitors). The substance is well tolerated by older patients (Goetz et al. 2006). There is discussion on potential interactions with foodstuffs containing teramine, but this appears to be merely a theoretical risk (deMarcaida et al. 2006; LeWitt 2009). A “serotonine effect” of rasagiline is also possible, but basically very improbable (Montgomery and Panisset 2009; Panisset et al. 2014); the STACCATO study at any rate found no such symptoms in 471 patients (Panisset et al. 2014). Patients in the ADAGIO study profited from a combination of an SSRI with rasagiline (Smith et al. 2015). Nonetheless, combining with SSRI requires some amount of caution.

Health economic evaluations indicate that rasagiline can be classified as beneficial (Farkouh et al. 2012; Haycox et al. 2009; Hudry et al. 2006; McCormack 2014).

## Safinamide

Safinamide was introduced to the market in 2015 and was at the time the first market launch for a specific Parkinson treatment for over 10 years. In principle, the substance can be categorized with drugs with a MAO-B inhibiting effect, but is treated as distinct because of the complex dual mechanism of action (Caccia et al. 2006; Olanow and Stocchi 2016), whose clinical importance we still cannot explain conclusively. As in the case of the two other MAO-B inhibitors, a neuroprotective capability is under discussion (Sadeghian et al. 2016).

### Pharmacology

Safinamide is an α-aminoamide derivate and has a MAO-B inhibiting effect and both a dopaminergic and a non-dopaminergic mode of action, by among others blocking the voltage-dependent sodium channels.

Safinamide blocks the voltage-dependent sodium and, to a lesser degree, the calcium channels (Olanow et al. 2016). Due to the application-dependent modulation of voltage-dependent sodium channels, the excessive release of glutamate is reduced without influencing the basal glutamate levels. The clinical significance of this lies in the fact that only the pathological glutamate release is arrested.

### Mode of action of safinamide

Safinamide is a highly selective, reversible MAO-B inhibitor which inhibits MAO-B in the human brain one thousand times more strongly than MAO-A. This MAO-B inhibition is exclusively pharmacodynamic, does not in any way induce structural modifications in the MAO-B enzyme and is thus completely reversible. There is a complete inhibition of platelet-derived MAO-B in the nanomolar range without any negative impact on MAO-A. Other dopaminergic mechanisms are either not influenced or at most only to a slight degree. Safinamide does not influence the enzymes that are involved in levodopa metabolism, such as aromatic l-amino acid decarboxylase (AADC) and catechol-*O*-methyltransferase (COMT).

The substance is water-soluble, demonstrates a high capacity for permeability and is quickly reabsorbed. The plasma–protein bond is approximately 90% and is not subject to a significant first-pass effect. There is dose linearity for *C*_max_ and AUC. The bioavailability is constant over all different doses. After administering 50 mg safinamide under fasting conditions, the absolute bioavailability of safinamide is high (on the average 95%) and after administering an individual dose of 100 mg, the maximum plasma concentration (*T*_max_) is reached after 2 to 2.5 h (*C*_max_ at approximately 650 ng/ml and AUC at 19.000 ng/ml × h) (Fariello 2007). The half-life period is about 24 h, a steady-state is dose-dependent with but minor interindividual variability (Marzo et al. 2004). The pharmacokinetics is not subject to influence from the variables of age, gender or race of the patient. The elimination half-life (*T*1/2) of safinamide amounts to approximately 20 to 24 h (Marzo et al. 2004).

Within a range of between 300 and 10 mg/kg, safinamide has linear pharmacokinetics. Under joint administration with levodopa and/or dopamine agonists, no effect could be seen on the clearance of safinamide, and the pharmacokinetic profile for the simultaneous application of levodopa was not altered (Borgohain et al. 2014b). The so-called “cocktail study” with CYP1A2 and CYP3A4 substrates (coffein and midazolam) showed no clinically significant effect on the pharmacokinetic profile of safinamide. Further studies demonstrated that CYP enzymes play only a subordinate role in the biotransformation of safinamide. Because safinamide can temporarily inhibit BCRP (Breast Cancer Resistance Protein), an interval of 5 h should be adhered to between administering safinamide and then giving medication that has a BCRP substrate with a Tmax of ≤ 2 h (such as pitavastatin, pravastatin, ciprofloxacin, methotrexate, topotecan, diclofenac or glyburide).

Safinamide is eliminated exclusively by being metabolised and subsequently it is predominantly eliminated in the urine. An extensive biotransformation leads to a slight elimination of the unmodified substance (approximately 2% in the faeces and 7% in the urine) (Onofrj et al. 2008). All essential metabolites (NW-1153, NW-1199 and NW-1689 glucuronide) are considered inactive as far as effectiveness and safety are concerned.

In the case of patients presenting with mild or moderate renal dysfunction, dosage adjustments are not necessary. In moderate liver dysfunction (Child–Pugh B), safinamide can increase exposition by ~ 80%. And patients with moderate curtailments in liver functioning require a lower dose (50 mg/day).

Safinamide does not have any clinically relevant influence on the depletion of tyramine (Cattaneo et al. 2003; Di Stefano and Rusca 2011; Marquet et al. 2012). 100 mg/day of safinamide reinforces the circulatory effects of oral tyramine vs. placebo by 1.6 times. A supra-therapeutic dose of 350 mg/day led to a 1.8 potentiation (Marquet et al. 2012). A possible potentiation of the hypertensive effect of oral tyramine by safinamide was studied in 20 healthy subjects who received safinamide at a dose of 300 mg/day for six to seven days (Di Stefano and Rusca 2011). Safinamide failed to show any clinically relevant tyramine-induced rise in blood pressure.

It is important to note that safinamide does not have any relevant risk for a substance-induced Torsade-de-pointes syndrome and thus has a favourable cardiac safety profile. There is no negative influence on the QT interval. In therapeutic (100 mg/day) as well as supra-therapeutic (350 mg/day) doses safinamide can in fact induce a slight dose-dependent shortening of the QT interval (~ 5 ms). The SETTLE and the MOTION studies did not show any increase in the QTc interval.

### Studies on safinamide with special clinical relevance

The first clinical studies suggest that safinamide might have an effect more than just MAO-B inhibition, because, under complete MAO-B inhibition through increase in dosage, further positive effects were observed on the fluctuations (Fariello 2007; Stocchi et al. 2006).

In further clinical studies, over 3000 patients were examined and over 500 patients were seen for a period of 2 years (Cattaneo et al. 2020; Olanow and Stocchi 2016). The most relevant studies here are: 016, 018, SETTLE, SYNAPSES and MOTION (Abbruzzese et al. 2021; Barone et al. 2013; Borgohain et al. 2014a, b, Schapira et al. 2013b, 2017).

In the phase III study 016 (Borgohain 2014a), effectivity and safety were examined in a double-blind, placebo-controlled parallel group study in Parkinson patients who were in intermediate to late stages with motor fluctuations and who before study begin had been receiving only levodopa or levodopa in combination with other Parkinson’s medications. In addition to their levodopa dosage, the patients were then given for 24 weeks either: 100 mg/day (*n* = 224), 50 mg/day safinamide (*n* = 223) or placebo (*n* = 222). The primary outcome was the change in on-time without disturbing dyskinesias (in the DRS: the Dyskinesia Rating Scale). At week 24, an improvement was found for the duration of on-time: by 1.36 ± 2.6 h for the 100 mg/day arm, by 1.37 ± 2.7 h for the 50 mg/day arm and by 0.97 ± 2.4 h for the placebo group. Significance was found for both the treatment arms. In addition, the secondary target parameters, off-time, UPDRS Part III and CGI-C, were also significantly improved in both arms of the study. There were no differences in the side effects in the three study groups. It is important to note that "placebo" here in this discussion did not mean that the patients were given a specific placebo substance but rather that they kept receiving their standard therapy without any additional safinamide.

The study was conducted as an extension study (018) (Borgohain et al. 2014b; Cattaneo et al. 2015). Dyskinesias made up the primary outcome as evaluated by the Dyskinesia Rating Scale. The placebo group consisted initially of 175 patients (142 at the end), 189 (148) were given 50 mg safinamide, 100 mg received 180 (150) patients. The participants remained in the same treatment group to which they had been randomised in Study 016. In both safinamide groups, a decrease in their DRS total score was seen compared to baseline:

- 31% reduction—Safinamide 50 mg/day
- 27% reduction—Safinamide 100 mg/day
- 3% reduction—placebo

The primary outcome was not in fact achieved, but at the time of the baseline measurements, the majority of the patients (64%) had either no or at most only mild dyskinesias (DRS ≤ 4), so that here no improvement was to be expected. Therefore, a post hoc analysis of the DRS data was performed for 242 patients who had already presented with moderate to severe dyskinesias at the start of the study 016 (DRS total score > 4). This time significance was clearly seen in the 100 mg arm (*p* = 0.0317) but not in the 50 mg arm (Cattaneo et al. 2015). This positive effect could be seen in all subgroups, even for all combinations of medications (Cattaneo et al. 2015). The improvement in on-time amounted to over one hour in both arms.

The SETTLE Study (Schapira et al. 2017) was initiated in 2009, and their data became decisive for the final approval of safinamide. The study design itself was in fact even included in the approval text and is to a considerable extent identical with that of Study 016. Between 2009 and 2012 Parkinson patients were assigned in equal numbers to either a safinamide or a placebo treatment group for a duration of 24 weeks. In the first two weeks, the patients received 50 mg safinamide and subsequently this dose was raised to 100 mg (in tablet form in all cases).

After reaching the optimized medication setting/dosage, the patients had to have at least 1.5 h of off-time daily (with the one exception of early morning off-time) and also had to have received levodopa (with a decarboxylase inhibitor) for at least 4 weeks in a fixed dose. 851 patients were examined as to their eligibility for being selected for the final study and thus 549 were found for randomization. 245 safinamide patients (89.4%) and 241 placebo-treated (87.6%) completed the course of the study. Due to adverse side effects, 12 patients in the safinamide group (4.4%) and 10 placebo-treated (3.6%) ones withdrew prematurely.

The primary outcome was the amount of improvement in on-time without any afflicting dyskinesias. In the safinamide group, on-time could be extended by 1.42 h (after having started with approximately nine hours, and 0.57 h in the placebo group).

The MOTION study (Barone et al. 2013) examined safinamide in combination with dopamine agonists. In the group with dopamine agonists (*n* = 66), 100 mg/day of safinamide significantly improved the UPDRS score (*p* = 0.0396). The 50 mg arm failed to show significant improvement (*p* = 0.2280).

The SYNAPSES study (Abbruzzese et al. 2021) aimed at checking the safety profile, particularly in older patients and those with psychiatric and other comorbidities. Altogether 1610 patients were enrolled in eight different European countries with 82.4% evaluable after 12 months. 25.1% of patients were > 75 years, 42.4% with psychiatric conditions, and 70.8% with relevant comorbidities. Clinically significant improvements were seen in the UPDRS motor score and in the UPDRS total score in ≥ 40% of patients. In the patient group > 75 years, no relevant differences were found for AEs frequency, severity or action taken. 13.6% experienced SAE vs 7.7% of younger patients. In the group with relevant comorbidities, 49.1% experienced AEs vs 37.8% of patients without comorbidities. 11.1% experienced SAE vs 4.6% of patients without comorbidities. In the patients with psychiatric conditions, no relevant differences were found for AEs and SAEs frequency, severity and action taken. 58% patients had a dose increase from 50 to 100 mg/day (decision of the physician), 6% patients had a dose decrease from 100 to 50 mg/day (by decision/wish of the patient). In summary, neither age, comorbidities, nor psychiatric conditions seem to have any relevant effect on its safety profile. The final conclusion of the EMA-Type II variation assessment report was that “The benefit-risk balance of safinamide remains positive.” Another recent trial confirmed the safety of safinamide when administered with SSRIs and SNRIs in PD depressed patients (Pérez-Torre et al. 2021). No cases of serotonin syndrome were recorded, even in the group of subjects who were taking opioids. The authors concluded that concomitant use of safinamide with antidepressant drugs seems to be safe and well tolerated, in the long-term as well.

The X-TRA study (Jost et al. 2018) observed 297 patients between 2015 and 2017 under everyday conditions ("real life") exclusively in Germany and thus constitutes the largest completed study of its kind on safinamide. The motor and non-motor symptoms as well as the quality of life improved while unknown adverse side effects have not yet been observed.

Recently, Guerra and co-workers demonstrated the positive effect of safinamide on the relative excess of glutamate in patients (Guerra et al. 2019). In another trial, Geroin and co-workers found that after 12 weeks of safinamide therapy, a significant improvement was noted in several scales for pain (King’s Pain Scale for Parkinson’s Disease, Brief Pain Inventory Intensity and Interference, and Numerical Rating Scale) and also in UPDRS III and IV, CGI, and PDQ39 (Geroin et al. 2020).

No significant changes in LEP complexes were observed. They concluded that safinamide may be effective for the management of pain in PD patients with motor fluctuations.

The so-called SAFINONMOTOR study was able to demonstrate positive effects on pain, sleep and mood (Grigoriou et al. 2021; Laban Deira et al. 2021, Santos Garcia et al. 2021a, b). The improvement seen on sleep which was confirmed by another study focused on Rapid Eye Movement (REM) sleep behaviour disorders (Plastino et al. 2021), exploring the clinical and video-polysomnographic changes occurred during safinamide treatment in 30 PD patients. Twenty-two of 30 patients reported clear improvement in symptoms during safinamide treatment, and 16 were absolutely free from clinical RBD-symptoms at the end of the treatment.

Goméz-Lopéz and co-workers investigate the effects of safinamide in patients with urinary problems, using the SCOPA-AUT-U scale, and found that the total score, as well as urgency, incontinence, frequency and nocturia subscales improved significantly after safinamide treatment. They concluded that, although the mechanism of this effect remains unknown, a nondopaminergic multimodal effect of the drug could be speculated on (Goméz-Lopéz et al. 2021).

At present, the so-called SUCCESS study (EUPASS Registry number 41428) is being conducted in five European countries as an observational study comparing safinamide, rasagiline and dopamine agonists/COMT inhibitors under real life conditions and measuring the effectiveness of the drugs on quality of life and healthcare resources consumption.

Further publications recently published show that for the category of mood a positive effect was also observed (Cattaneo et al. 2017b) and to be exact in the scores in both the PDQ-39 and in the GRID-HAMID (Hamilton Rating Scale for Depression).

### Joint interpretation of the 016 and SETTLE studies

One interesting aspect stems from a post hoc analysis of the 016 and SETTLE studies (Cattaneo et al. 2017a, 2018). Because they have the same study design, 016 and SETTLE can readily be juxtaposed for comparison. In this analysis, the symptom of pain in the Parkinson’s Disease Quality of Life Questionnaire (PDQ-39) was specifically selected for review after six months into the study. In the safinamide, 100 mg arm fewer patients received an analgesic than in the comparison group. In week 24, a mere 23.9% of patients in the safinamide group and 30% of the placebo group were taking the pain reliever. Specifically reviewing individual parameters evaluated in the PDF-39, a distinct improvement can be seen in, for example, muscle cramps (item 37), joint pains (item 38) and unpleasant hot or cold sensations (item 39), and in the case of items 37 and 39 this difference is significant. Further clinical studies on pain symptoms have been initiated, some have already been concluded and confirm the positive effect (Grigoriou et al. 2021; Santos Garcia et al. 2021a, b).

## Comparison of the MAO-B inhibitors

The question as to whether differences exist between selegiline and rasagiline might be the case is best responded to by referring to the fact that they are pharmacologically different medications (LeWitt 2009; Müller and Reichman 2012). The power of the inhibition for both MAO-A and MAO-B is clearly different and, to be exact, by either two powers of ten resp. five-fold (Müller and Reichmann 2012). The bioavailability is different as well (selegiline < 10%) and only the pharmacokinetics of rasagiline is linear in the therapeutic range (Müller and Reichmann 2012). The profile of side effects reveals distinct advantages for rasagiline (Csoti et al. 2012; Müller and Reichmann 2012). On this point the degradation of selegiline to amphetamine and metamphetamine should be recalled (Müller and Reichmann 2012).

The present assessment of the Movement Disorder Society (Fox et al. 2018) again reveals distinct differences, for example in the combination therapy with levodopa with motor complications (rasagiline is effective, but there is insufficient evidence on selegiline). A recent evaluation found a slight superiority for selegiline compared to the other MAO-B inhibitors available on the market (Binde et al. 2018).

Unfortunately, there are no head-to-head studies here. In one smaller study (*n* = 28) selegiline was switched to rasagiline. All examined parameters revealed improvement (Jost et al. 2008). A further switch study (Müller et al. 2013) found superiority for rasagiline. In a so-called retrospective head-to-head comparison, no relevant differences were found between selegiline and rasagiline but rather lower levodopa doses and fewer dyskinesias (Cereal et al. 2017).

To compare the symptomatic effects, we did a meta-analysis in which the 6 RCT (randomized, controlled studies) with rasagiline and the RCT 15 with selegiline were included (Jost et al. 2012). In monotherapy, there were significant differences favoring rasagiline in UPDRS total scores and in the motor score. The meta-analysis of all studies (mono- and combination therapy) confirmed significant differences in favor of rasagiline in the UPDRS total score.

In studies on rasagiline, the termination rate due to adverse reactions was on the level after placebo treatment while the rate for selegiline was significantly higher than for placebos (Jost et al. 2012).

Peretz et al. (2016) analysed the necessity for dopaminergic medication under the two substances in 349 (selegiline) vs. 485 (rasagiline) patients. Both groups received levodopa after approximately three years. In the selegiline group, dopamine agonists were introduced at a later time. The data supported the view that selegiline had a slightly better symptomatic effect (Peretz et al. 2016).

So far there are no comparative studies of rasagiline or selegiline with safinamide. The so-called SUCCESS study could close this gap somewhat.

## Disease modification

No other substance has been examined as to the question of neuroprotection as early and as controversially as selegiline. At the present, there is no proof for either a relevant neuroprotective effect or a progression-delimiting effect (Fox et al. 2018; Schapira 2011). Nonetheless there are distinct theoretical as well as clinical indications for a disease-modifying effect (Marras et al. 2005; Naoi et al. 2020; Olanow et al. 1995; Riederer et al. 2018; Tábi et al. 2020). For example, the NET-PD LS1 study was able to demonstrate a retarded progression in the MAO-B inhibitor group.

The first speculations on a neuroprotective effect go as far back as Birkmayer, who found a lower rate of mortality among the patients in his selegiline group (Birkmayer et al. 1985). The fundamental explanation for this theoretical concept is the inhibition of oxidative stress in the dopaminergic neurones still intact, meaning that the concept is a model (the radikal hypothesis, H_2_O_2_). It is also a matter of discussion whether there is a neurotrophic and an anti-apoptotic effect [Naoi 78]. In the final analysis, however, the neuroprotective effect has only been demonstrated in the animal model, whereby in such models un-physiological conditions prevailed as for example in the MPTP model (neurotoxine hypothesis).

One other indication of a neuroprotective action can be seen in the quick recovery of patients after a cerebrovascular accident if they are treated with selegiline (Sivenius et al. 2001).

The frequently cited DATATOP study [PSG 1989a; PSG 1989b; PSG 1993; PSG 1998] has only limited use for supporting this neuroprotective effect because (1) on the one hand delaying the need for levodopa could be a purely symptomatic effect and (2) on the other hand even a renewed evaluation of this study’s results showed that there were no long-term relevant differences. We have to assume that the delayed use of levodopa by as much as several months is a result of the symptomatic effect of selegiline and not an inherently potentially neuroprotective capacity. And so no differences were found in the long-term course of the disease with or without selegiline.

Furthermore, a remark is in place here about selegiline being tested against vitamin E which has been reputed to have a neuroprotective effect and has in fact been supported in studies (de Rijk et al. 1997). Furthermore, in the DATATOP study, the above-average life expectancy of the patients was striking (PSG 1998), which stands in contradiction to other studies (Hely et al. 1999).

A contrary view on the effect of selegiline was published by a British workgroup (Lees 1995). In this particular study, no relevant symptomatic effect could be found and the mortality rate was in fact greater than in the control group. However, the study is debatable primarily due to its having a suboptimal study protocol (and the far-reaching conclusions of the publication should also be evaluated critically [31]). But their results nonetheless strongly unsettled any belief in a neuroprotective effect of selegiline permanently. Reviewing this study (Lees 1995), it is often overlooked that no routine levodopa increase had been required, and that could argue for a neuroprotective capacity. A publication by Margaret Thorogood relativized the assertions of the Lees team, but still came to the conclusion that mortality was discretely higher in the patients treated with selegiline than in levodopa monotherapy and in particular in patients with a younger age at onset (Thorogood et al. 1998). In the meantime, the data from the Lees study (Lees 1995) can be seen as sufficiently refuted (Marras et al. 2005).

A number of studies, for example by Olanow et al. (1995), demonstrated a slower rate of progression, as for example in the renewed analysis of the DATATOP study (PSG 1998). The question of a neuroprotective impact was again addressed in the study by Pålhagen et al. (2006) in a placebo-controlled study of 140 patients covering altogether 7 years. After 5 years, the UPDRS total score showed a difference of 28.7 ± 14.7 (selegiline) versus 38.6 ± 15.5 (placebo) and a difference in the levodopa dose 529 ± 145.6 mg (selegiline) versus 631 ± 186.3 mg (rasagiline), thus signifying an improvement in the UPDRS score of ten points and at the same time savings in levodopa of approximately 100 mg (Pålhagen et al. 2006).

A neuroprotective effect of rasagiline has also been discussed and was demonstrated in several studies (Finberg et al. 1996; Jenner 2012; Marras et al. 2005; Schulz 2012; Youdim et al. 2005). In particular, aminoindane may be playing a role here because, as opposed to amphetamine, it has potentially neuroprotective characteristics (Bar-Am and Amit 2004; Müller and Reichmann 2012). This possible neuroprotective potency was confirmed in a clinical study (PSG 2004) in patients with early onset who had a better course than patients whose therapy was delayed by 6 months. The ADAGIO study (Schapira et al. 2013a) furthermore demonstrated a disease-modifying effect at least for the 1 mg group of patients. This has not been found for any other substance. Unfortunately in the follow-up time, the two groups conformed more and more to each other (Rascol et al. 2016). The PROUD study, which had the same design using pramipexole, was negative, thus making the claims of the ADAGIO study all the more positive (Schapira et al. 2013a). But fundamentally it should be emphasized that all studies are initiated well into the course of the disease, that is, much too late as to ever show a clinically observable effect in neuroprotection (Naoi et al. 2020).

## Further MAO-B inhibitors and the future of MAO-B inhibitors

In addition to selegiline, various other MAO-B inhibitors have found clinical use. Mention should be made of, for example: lazabemide, milacemide and mofegiline. The clinical studies were discontinued in the majority of the substances being tested (e.g. mofegilin). Further MAO-B inhibitors are in development (Binda et al. 2006; Yelekçi et al. 2013).

Even after four decades of clinical use of MAO-B inhibitors, there are still a lot of unsolved questions. Because rasagiline and selegiline are generic medications, however, larger clinical studies are no longer now being undertaken and the evidence from earlier studies on selegiline do not meet present-day standards. The potential in MAO-B inhibitors has definitely not yet been fully exhausted (Tábi et al. 2020). The topic of neuroprotection might very well once again gain momentum, because MAO-B inhibition modulates α-syn secretion and aggregation (Nakamura et al. 2021). There is the good possibility that a new patch application for rasagiline will be developed, which then poses the question as to just which clinical use this would have for Parkinson therapy (Bali and Salve 2020). Considerable hope can be placed especially in safinamide inasmuch as clinical studies are underway and because new approaches for non-motor dysfunctions can develop out of the dual approach (Pagonabarraga et al. 2021).
